# Functional Characterization of Temporin-SHe, a New Broad-Spectrum Antibacterial and Leishmanicidal Temporin-SH Paralog from the Sahara Frog (*Pelophylax saharicus*)

**DOI:** 10.3390/ijms21186713

**Published:** 2020-09-13

**Authors:** Sonia André, Zahid Raja, Vincent Humblot, Christophe Piesse, Thierry Foulon, Denis Sereno, Bruno Oury, Ali Ladram

**Affiliations:** 1 CNRS, Institut de Biologie Paris-Seine, IBPS, BIOSIPE, Sorbonne Université, F-75252 Paris, France; smc.andre88@gmail.com (S.A.); zahid.rj@gmail.com (Z.R.); thierry.foulon@sorbonne-universite.fr (T.F.); 2 Cell death in host-pathogen interactions, CNRS-ERL3649, Université de Paris, 75006 Paris, France; 3 FEMTO-ST Institute, UMR CNRS 6174, Université Bourgogne Franche-Comté, 25030 Besançon CEDEX, France; vincent.humblot@femto-st.fr; 4 CNRS, Institut de Biologie Paris-Seine, IBPS, Peptide Synthesis Facility, Sorbonne Université, F-75252 Paris, France; christophe.piesse@sorbonne-universite.fr; 5 IRD, Université de Montpellier, MiVegec, 34394 Montpellier CEDEX 5, France; denis.sereno@ird.fr; 6 IRD, Université de Montpellier, InterTryp, 34394 Montpellier CEDEX 5, France; bruno.oury@ird.fr

**Keywords:** frog antimicrobial peptide, temporin-SHe, broad-spectrum activity, bacteria, parasites, secondary structure, membrane disrupting mechanism, scanning electron microscopy

## Abstract

Amphibian skin is a promising natural resource for antimicrobial peptides (AMPs), key effectors of innate immunity with attractive therapeutic potential to fight antibiotic-resistant pathogens. Our previous studies showed that the skin of the Sahara Frog (*Pelophylax saharicus*) contains broad-spectrum AMPs of the temporin family, named temporins-SH. Here, we focused our study on temporin-SHe, a temporin-SHd paralog that we have previously identified in this frog but was never structurally and functionally characterized. We synthesized and determined the structure of temporin-SHe. This non-amphipathic α-helical peptide was demonstrated to strongly destabilize the lipid chain packing of anionic multilamellar vesicles mimicking bacterial membranes. Investigation of the antimicrobial activity revealed that temporin-SHe targets Gram-negative and Gram-positive bacteria, including clinical isolates of multi-resistant *Staphylococcus aureus* strains. Temporin-SHe exhibited also antiparasitic activity toward different *Leishmania* species responsible for visceral leishmaniasis, as well as cutaneous and mucocutaneous forms. Functional assays revealed that temporin-SHe exerts bactericidal effects with membrane depolarization and permeabilization, via a membranolytic mechanism observed by scanning electron microscopy. Temporin-SHe represents a new member of the very limited group of antiparasitic temporins/AMPs. Despite its cytotoxicity, it is nevertheless an interesting tool to study the AMP antiparasitic mechanism and design new antibacterial/antiparasitic agents.

## 1. Introduction

In the age of resistance to conventional antibiotics, antimicrobial peptides (AMPs) appeared as promising tools against multidrug-resistant pathogens [1]. Among these key effectors of the innate immune system, and those contained in frog skin secretions are of particular interest considering their broad-spectrum antibacterial, antifungal and antiparasitic activities, and also their therapeutic potential [2,3,4,5,6]. Temporins represent an abundant family of small hydrophobic α-helical AMPs, C-terminally amidated, and bearing a low positive net charge (0 to +3) [7,8,9]. These peptides are found in the skin of Eurasian and New World ranid frogs. Being mainly active against Gram-positive bacteria, including antibiotic-resistant strains [10,11,12], only a few temporins are able to target Gram-negative bacteria [13,14,15,16,17], parasites [13,14,15,18,19] and viruses [20,21,22].

We have previously identified several new members of the temporin family, named temporins-SH, isolated from the ranid frog *Pelophylax saharicus* (also called Sahara Frog, North African Frog or Sahara Green Frog) (Table 1).

Despite their paralogous relationships, temporins-SH differ in their physicochemical properties (Table 1). Temporins-SH can be gathered into three groups comprising 13, 16–17 and 8 amino acid residues (Table 1). Alignment of the 13-residue temporins, SHa, SHb and SHc, reveals differences in their sequences. Temporin-SHa shares 61.5% identity with temporin-SHc, while only 31% identity is observed with temporin-SHb. As indicated in Table 1, temporin-SHc has a lower hydrophobicity (GRAVY = 1.34) compared to temporins SHa and SHb. Temporin-SHd and temporin-SHe belong to the long temporin subfamily with their 17 and 16 residues, respectively (Table 1). Temporin-SHd has only an additional glycine residue when compared to temporin-SHe, thus sharing the highest identity (76.5%) among all the temporins-SH. Moreover, temporin-SHd is slightly less hydrophobic than temporin-SHe (GRAVY: 1.65 and 1.78, respectively). With its eight residues only, temporin-SHf is considered as the smallest temporin and linear AMP found in nature (Table 1). This atypical ultra-short temporin is a highly hydrophobic and Phe-rich peptide (75% hydrophobicity, 50% of phenylalanine residues).

Temporins adopt an α-helical structure in apolar or membrane-mimicking environments, as well as in the cellular environment [8,11,15,23,25,26]. A well-defined amphipathic α-helical structure is observed for temporins SHa, SHb and SHc, with segregation of hydrophobic and hydrophilic/basic residues between the two opposite faces of the helix [23]. In contrast, temporin-SHd adopts an α-helical structure with a “polar” face not as completely segregated, mainly composed of neutral glycine and small apolar residues (alanine and proline) in addition to the lysine residue [15]. As it is the case for temporins, this amphipathic α-helical structure enables temporins-SH to disrupt the target microorganism membrane through a “carpet-like” membranolytic mechanism [8,9,14,15,27,28].

Temporins-SH differ in their antimicrobial activities. Temporin-SHc has a more classic temporin spectrum, targeting Gram-positive bacteria and yeasts/fungi. In contrast, the temporin-SHb paralog, bearing the same positive net charge (+1), has weak activity against the Gram-positive *Staphylococcus aureus* and *Bacillus megaterium*, and against the fungus *Aspergillus flavus*. Despite its short size, temporin-SHf was found to be active toward Gram-positive bacteria and toward the Gram-negative *Escherichia coli* [8]. Potent temporin-SHf analogs were obtained by combining natural and unnatural amino acid substitutions [29] or by converting the phenylalanine-rich temporin-SHf into tryptophan-rich peptides [30]. These analogs were able to affect a wider range of clinically relevant Gram-negative bacteria while retaining the non-cytotoxic character of the parent peptide [29], or to target *Candida* species [30]. Among temporins-SH, temporin-SHa is undoubtedly the most potent and broad-spectrum AMP with antibacterial, antifungal, antiparasitic and antiviral activities [14,22,31]. In addition, following covalent immobilization onto gold surfaces, this peptide retains its antimicrobial activity [32]. Temporin-SHd is also considered as a broad-spectrum temporin-SH because it is able to target Gram-positive and Gram-negative bacteria, and trypanosomatid parasites [15].

Considering that temporins-SH of the Sahara Frog are interesting small peptides that could serve as tool/template to decipher the antiparasitic/antiviral mechanism of AMPs or design new optimized compounds with therapeutic potential, we focused on temporin-SHe, a 16-residue peptide previously identified in this frog but never characterized (Table 1). In this study, the structural and functional characterization of temporin-SHe was undertaken in comparison to its most closely related paralog, temporin-SHd. We analyzed the structural conformation of temporin-SHe by circular dichroism in interaction with membrane mimicking environments. We also examined the antimicrobial activity against a wide range of microorganisms (bacteria, yeasts/fungi, *Leishmania* parasites) and antibiotic-resistant strains of *S. aureus*, and its toxicity on mammalian cells. The mechanism of action of temporin-SHe was confirmed on bacterial model membrane vesicles, using differential scanning calorimetry, and on bacterial cells by performing membrane depolarization/permeabilization and time–kill assays, and also scanning electron microscopy imaging. The results indicate that temporin-SHe represents a broad-spectrum antibacterial/antiparasitic temporin-SH. Like its paralogs temporin-SHa and temporin-SHd, this peptide could be used as a tool for the analysis of the antiparasitic mechanism of temporins, and also as a template for peptide-based strategies against bacterial and parasitic infections.

## 2. Results

### 2.1. Conformational Study

**Figure 1:**
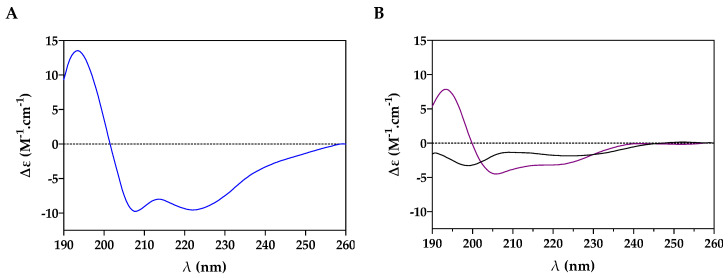
Secondary structure of temporin-SHe in the membrane-mimicking environment. (**A**) Circular dichroism (CD) spectrum of temporin-SHe in PBS containing negatively charged DMPC/DMPG (3:1) LUVs (1 mg/mL) at a peptide/lipid molar ratio of 1:100. (**B**) CD spectrum of temporin-SHe (30 µM) in sodium dodecyl sulfate (SDS) 80 mM (purple line) and PBS (black line). The CD signal corresponds to the dichroic increment (∆ε) per residue.

The secondary structure of temporin-SHe was determined by performing circular dichroism experiments with the synthetic peptide. In a bacterial membrane-mimicking environment, corresponding to negatively charged DMPC/DMPG (3:1) large unilamellar vesicles (LUVs), an α-helical ordered structure was observed for synthetic temporin-SHe with the two characteristic minima at 208 and 222 nm (Figure 1A). An α-helical structure was also observed in SDS micelles, whereas in aqueous solution (PBS) temporin-SHe demonstrated a random coil spectrum (Figure 1B). 

As it was previously observed for temporin-SHd 30 µM [15], temporin-SHe displayed a much lesser minimum intensity at 222 nm in 80 mM SDS compared to DMPC/DMPG LUVs. The percentage of α-helical content (−∆ε_222nm_ per residue × 10) was higher (95.5%) when temporin-SHe was bound to DMPC/DMPG LUVs (31% only in SDS micelles). This was also observed for temporin-SHd (82% of α-helical structure in DMPC/DMPG LUVs and 32% in SDS micelles) [15]. Another interesting point is the 222/208 nm amplitude ratio, which is a diagnostic of coiled-coil structure or non-interacting structure (ratio around 0.8 for single-stranded helices; ratio around 1 for a two-stranded helical coiled-coil). As revealed by the ratio values, temporin-SHe is structured as a two-stranded helical coiled-coil in DMPC/DMPG LUVs (ratio = 0.98) while as single-stranded non-interacting helices in SDS micelles (ratio = 0.74), like temporin-SHd [15].

**Figure 2:**
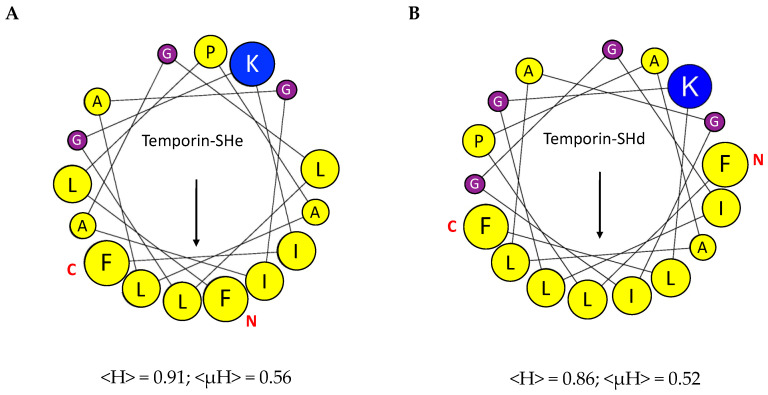
Schiffer–Edmundson helical wheel projection. (**A**) Temporins SHe. (**B**) Temporin-SHd. Apolar residues are colored in yellow. Neutral Gly residues appear in purple and basic residues in blue. Amino acid residues are represented proportionally to their volume. N and C letters in red indicate N- and C-peptide termini. The hydrophobic moment vector is denoted by an arrow. The hydrophobicity (<H>) and the hydrophobic moment (<µH>) are indicated.

Schiffer–Edmundson helical wheel projections of temporin-SHe and its paralog temporin-SHd revealed, for both peptides, a relatively low amphipathic character with no well-separated polar and apolar faces (Figure 2). One face of the helix is highly hydrophobic and delimited either by leucine (Figure 2A) or phenylalanine (Figure 2B) residues for temporin-SHe and temporin-SHd, respectively. The other “polar“ face of the two peptide α-helix is bordered by neutral glycine residues and constituted of a single basic residue (Lys) with alternated glycine and small apolar (proline and alanine) residues. This relatively low observed amphiphilicity is in agreement with the values of the helical hydrophobic moment (<µH>) of temporin-SHe and temporin-SHd compared to the hydrophobicity values (<H>) (Figure 2).

### 2.2. Interaction of Temporin-SHe with a Bacterial Membrane Model

Since we observed an α-helical structuration when temporin-SHe was bound to negatively charged DMPC/DMPG vesicles (bacterial membrane model), we next used differential scanning calorimetry (DSC) to assess the thermal transitions in DMPC/DMPG 3:1 multilamellar vesicles (MLVs) upon addition of temporin-SHe. Different peptide/lipid molar ratios (1:200, 1:100 and 1:50) were used by adding different concentrations of temporin-SHe once MLVs were formed.

**Figure 3:**
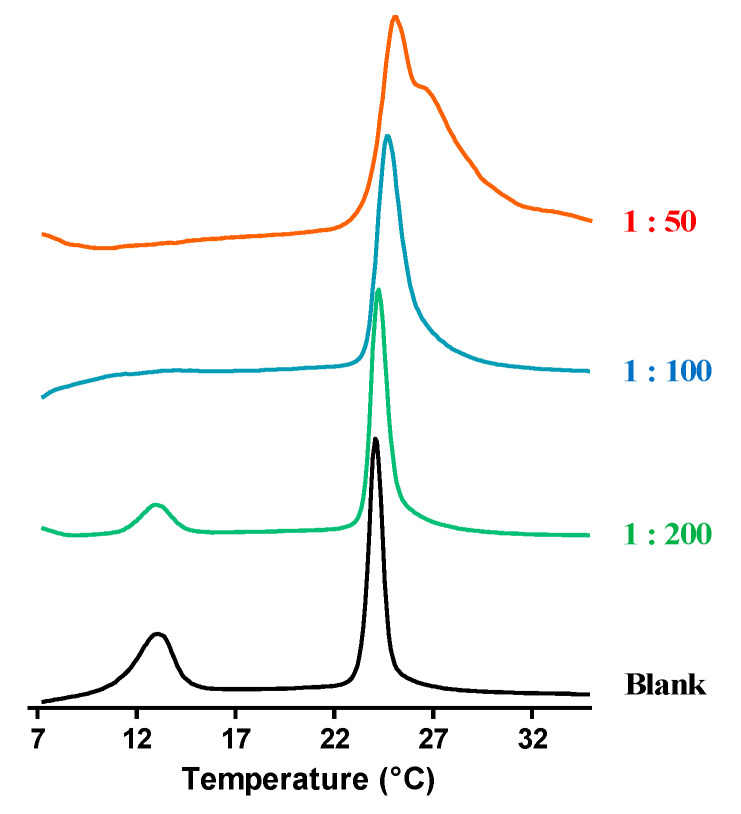
DSC heating thermograms illustrating the effect of temporin-SHe on the thermotropic phase behavior of DMPC/DMPG 3:1 multilamellar vesicles (MLVs). Scans were acquired with no peptide (blank) and at different peptide/lipid molar ratios (1:200, 1:100 and 1:50).

MLVs alone exhibited two endothermic peaks on heating (Figure 3), one near 13 °C (weakly energetic pretransition) and the other around 23–24 °C (strongly energetic main transition). These transitions are in agreement with those previously reported [23,33]. In the presence of temporin-SHe, the pretransition peak was reduced at peptide–lipid ratio 1:200, and abolished at 1:100 and 1:50 ratios. Since the pretransition is due to interactions between the phospholipid headgroups, this indicates a significant alteration of these interactions due to electrostatic interactions between the cationic temporin-SHe and anionic lipid headgroups. Temporin-SHe affected also the main transition peak. Indeed, a noticeable change in the shape of this peak is observed with increasing peptide concentration, leading to a two-component main phase transition at peptide–lipid ratio 1:50 (Figure 3). A two- or multicomponent main phase transition was previously reported for temporins-SH [15,23]. This suggests that temporin-SHe disturbs strongly the membrane bilayer by affecting hydrocarbon chain packing, with the two components corresponding to peptide-poor and peptide-rich phospholipid domains.

### 2.3. Antimicrobial Activities

The antimicrobial activity of synthetic temporin-SHe was assessed against various Gram-positive (*S. aureus* strains, *Enterococcus faecalis*, *B. megaterium* and *Listeria ivanovii*), and Gram-negative bacteria (*E. coli* strains, *Salmonella enterica*, *Pseudomonas aeruginosa*, *Acinetobacter baumannii* and *Klebsiella pneumoniae*), and yeasts/fungi (*Candida albicans*, *C. parapsilosis* and *Saccharomyces cerevisiae*) (Table 2).

Temporin-SHe was highly active against all tested Gram-positive bacteria (MIC range, 1.56 to 12.5 µM), including the antibiotic-resistant *S. aureus* ATCC 43300 strain and the multidrug-resistant *S. aureus* ATCC BAA-44 strain. It was also efficient against Gram-negative bacteria, such as *E. coli* ATCC 25922 and *A. baumannii* ATCC 19606 (MIC = 25 µM), and moderately active against other *E. coli* strains and *P. aeruginosa*. In contrast, temporin-SHe was virtually inactive against *K. pneumoniae* and *S. enterica*. Compared to temporin-SHe, the antimicrobial spectrum of temporin-SHd was overall quite similar—with, however, as significant differences—a five-fold higher activity against the *E. coli* ATCC 25922 strain and no activity against *P. aeruginosa* and *Candida parapsilosis* (Table 2).

**Figure 4:**
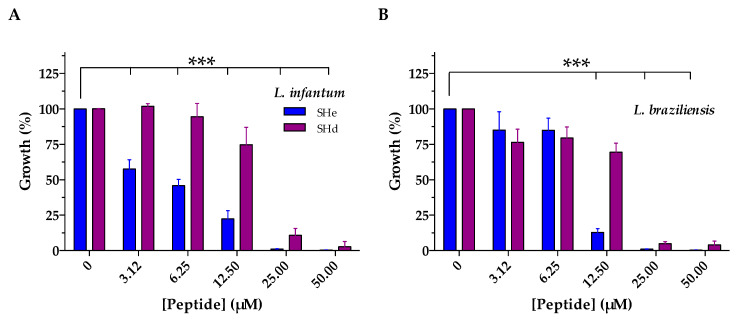

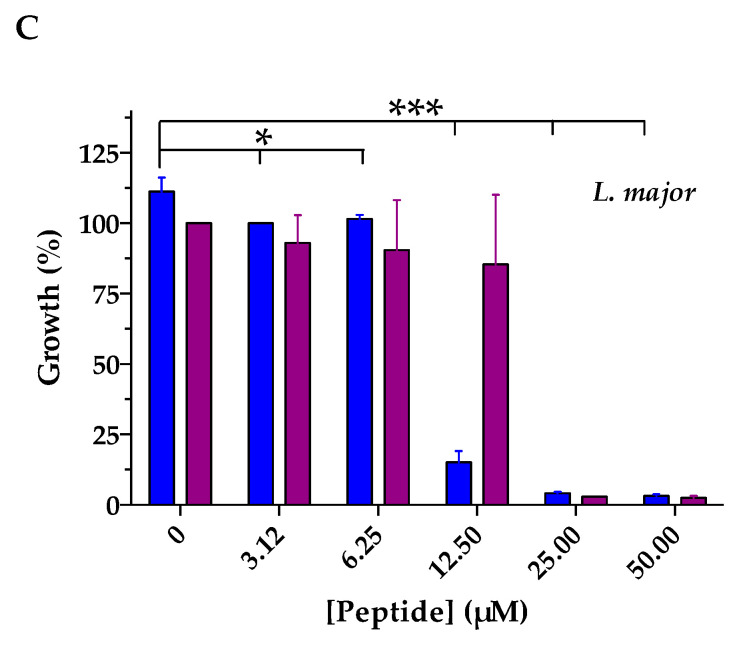
Dose-dependent leishmanicidal effects of temporin-SHe and temporin-SHd against different species of *Leishmania* promastigotes. (**A**) *L. infantum*. (**B**) *L. braziliensis*. (**C**) *L. major*. Data are the mean ± S.D. of three independent assays performed in triplicates. For temporin-SHd, data were taken from previous experiments [15] and presented as histograms for comparison with temporin-SHe. Statistical differences were assessed for temporin-SHe using one-way ANOVA followed by Dunnett’s test (* *p* < 0.05; ** *p* < 0.01; *** *p* < 0.001).

Considering the fact that temporin-SHd is a very close paralog of temporin-SHe and that it was previously shown to be active against trypanosomatid parasites [15], we evaluated the antiparasitic activity of temporin-SHe against different species of *Leishmania* parasites (Figure 4, Table 3). Temporin-SHe demonstrated dose-dependent anti-*Leishmania* effects against promastigote forms (insect stage) of *Leishmania infantum*, *L. braziliensis*, and *L. major* (Figure 4). At the lowest peptide concentration tested (3.12 µM), 42% inhibition of *L. infantum* growth was observed after incubation with temporin-SHe, whereas no effect was observed with temporin-SHd at this concentration (Figure 4A). Temporin-SHe was less active against *L. braziliensis* (Figure 4B) and *L. major* (Figure 4C) promastigotes compared to *L. infantum* but was more potent than temporin-SHd. Indeed, *Leishmania* growth inhibition was significant and much greater at 12.5 µM for temporin-SHe, with a temporin-SHe-induced inhibition of 87% (temporin-SHd: 31%) and 85% (temporin-SHd: 15%) for *L. braziliensis* and *L. major*, respectively.

Mean IC_50_ values were determined and revealed that temporin-SHe was highly potent against *L. infantum* (IC_50_ = 4.6 µM), with enhanced leishmanicidal activity compared to its paralog temporin-SHd (Table 3).

### 2.4. Cytotoxic Activities

**Figure 5:**
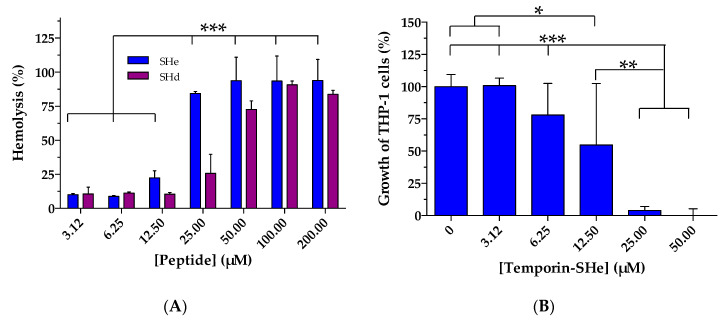
Toxic activity of temporin-SHe against mammalian cells. (**A**) Comparison of temporin-SHe and temporin-SHd dose-dependent effects on human erythrocytes. Percent hemolysis was calculated by normalizing to PBS-treated cells (0% hemolysis) and Triton X-100-treated cells (100% hemolysis). (**B**) Effect of temporin-SHe on human THP-1 monocytes. Data are the mean ± S.D. of two independent assays performed in triplicates. Statistical differences were assessed for temporin-SHe using one-way ANOVA followed by Tukey’s multiple comparison test (* *p* < 0.05; ** *p* < 0.01; *** *p* < 0.001).

The hemolytic properties of temporin-SHe against human erythrocytes were evaluated in comparison to those of temporin-SHd (Figure 5A). Temporin-SHe was found to be much hemolytic than temporin-SHd as 84% hemolysis was obtained as soon as a concentration of 25 µM (22% hemolysis at 12.5 µM), whereas 100 µM was needed for temporin-SHd to reach approximately (91%) this percentage (25% and 72% hemolysis at 25 µM and 50 µM, respectively). When hemolysis percentage was plotted against the log_10_ peptide concentration, mean LC_50_ (lytic concentration 50) values of 17 µM and 42 µM were determined for temporin-SHe and temporin-SHd, respectively. This more toxic character of temporin-SHe was further confirmed on the human leukemia monocyte cell line THP-1. Figure 5B indicates that 55% of viability was observed at a concentration of 12.5 µM temporin-SHe and only 4% at 25 µM. In addition, the calculated mean LC_50_ (lethal concentration 50) obtained from dose–response curves was 11.4 µM compared to temporin-SHd (LC_50_ = 66 µM, taken from reference [15]).

### 2.5. Alteration of Bacterial Membranes

**Figure 6:**
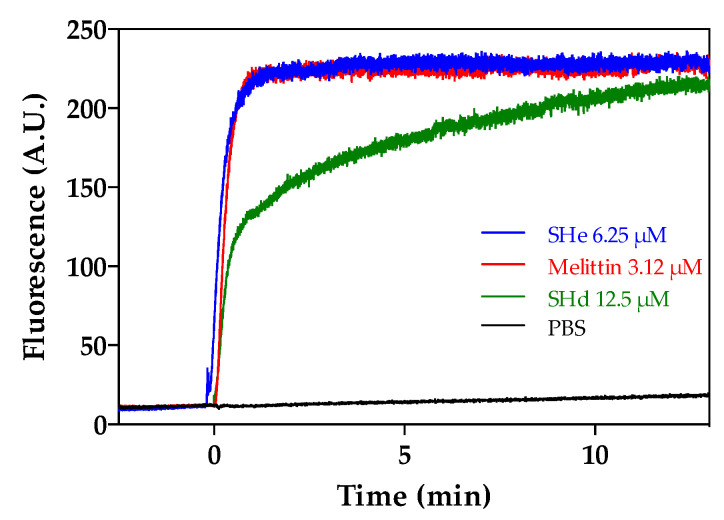
Temporin-SHe-induced membrane depolarization. The cytoplasmic membrane depolarization of *S. aureus* ATCC 25923 was monitored using the potentiometric fluorescent dye DiSC_3_(5). After equilibration with DiSC_3_(5), peptides were added (t = 0). Melittin was used as positive control, and PBS as negative control. The data shown are from a single experiment representative of three independent assays. A.U.: Arbitrary units.

The ability of temporin-SHe and temporin-SHd to alter the membrane potential of *S. aureus* ATCC 25923 bacteria was studied by monitoring the increase of fluorescence of the cationic lipophilic dye DiSC_3_(5) upon addition of peptides (Figure 6). Both peptides were able to dissipate the bacterial membrane potential, but temporin-SHe-induced depolarization was instantaneous (maximal threshold reached in the first minute) and to a higher extent than its paralog. The effect of temporin-SHe was comparable to that of melittin, a bee venom AMP used as positive control and known to induce pore formation [34].

**Figure 7:**
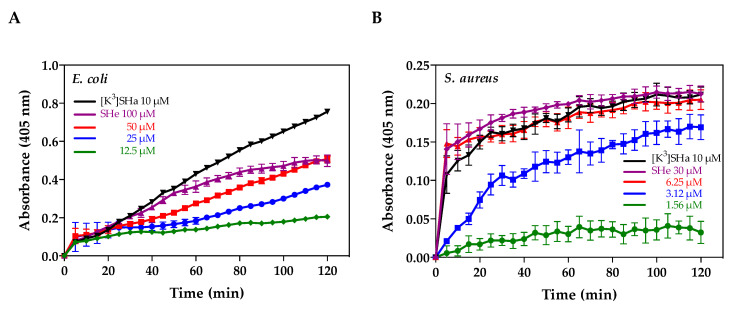
Time-dependent bacterial membrane permeabilization after treatment with increasing concentrations of temporin-SHe. (**A**) *E. coli* ML-35p. (**B**) *S. aureus* ST1065. [K^3^]-temporin-SHa (10 µM) was used as positive control (black lines). *o*-nitro-phenol (ONP) production was monitored by measuring absorbance at 405 nm. Data are from a representative experiment out of two experiments carried out in triplicates. They are expressed as the mean ± S.D. after subtraction of the negative control values (no peptide) from the test values.

Membrane permeabilization of the Gram-negative *E. coli* ML-35p and Gram-positive *S. aureus* ST1065 was also investigated by monitoring the hydrolysis of the chromogenic extracellular substrate *o*-nitro-phenyl-*β*-d-galactopyranoside (ONPG) into *o*-nitro-phenol (ONP) by bacterial cytoplasmic *β*-galactosidase. Temporin-SHe permeabilized cytoplasmic membranes of both bacteria in a time- and concentration-dependent manner (Figure 7). Taking into account temporin-SHe concentrations and the positive control [K^3^]-temporin-SHa (a potent temporin-SHa analog [14]), *S. aureus* bacteria (Figure 7B) appeared to be faster and more efficiently permeabilized than *E. coli* (Figure 7B).

### 2.6. Bacterial Killing

**Figure 8:**
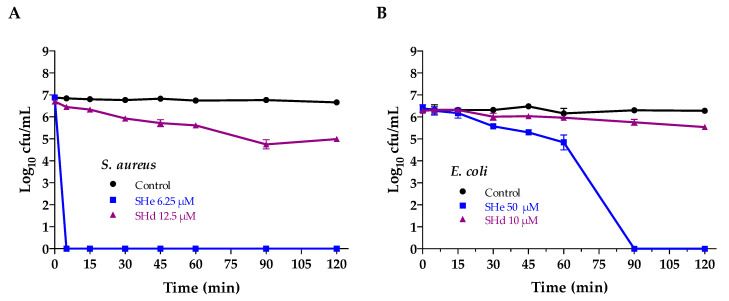
Comparison of the bactericidal effects of temporin-SHe and temporin-SHd at two-fold MIC concentration. (**A**) Effect on the Gram-positive *S. aureus* ST1065 after time-dependent incubation with 6.25 µM temporin-SHe and 12.5 µM temporin-SHd. (**B**) Effect on the Gram-negative *E. coli* ATCC 25922 after incubation with 50 µM temporin-SHe and 10 µM temporin-SHd. The control corresponds to bacteria incubated in PBS with no peptide. The data are the mean ± S.D. of one representative experiment out of two independent assays performed in triplicates.

Time–kill kinetics were performed by incubating the Gram-positive *S. aureus* (ST1065 strain) and the Gram-negative *E. coli* (ATCC 25922 strain) at different times with temporin-SHe or temporin-SHd at two-fold MIC concentration (Figure 8). Temporin-SHe showed a rapid potent killing effect on *S. aureus*, causing complete killing within the first 5 min (Figure 8A). A slower killing effect was observed on *E. coli*. (total killing at 90 min) (Figure 8B). Temporin-SHd was less effective than temporin-SHe. Indeed, only 1.7 log_10_ and 0.75 log_10_ reductions were obtained after 120 min incubation for *S. aureus* and *E. coli*, respectively.

### 2.7. Visualization of the Membranolytic Effect of Temporin-SHe on S. aureus Bacteria

**Figure 9:**
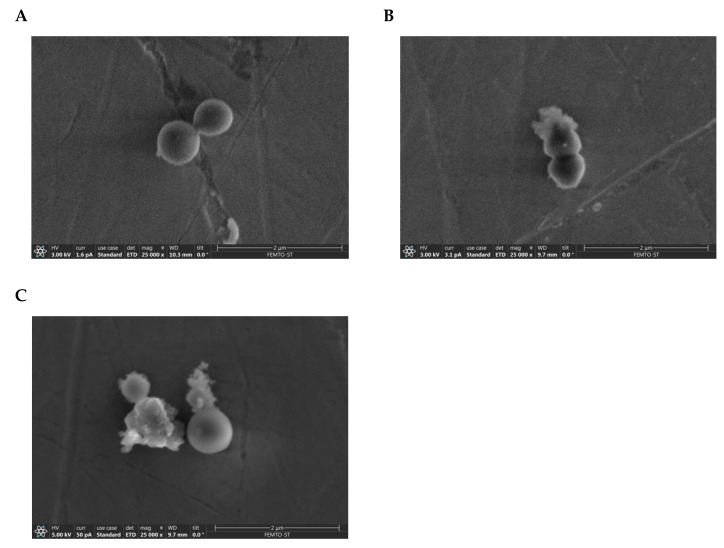
Scanning electron microscopy imaging of *S. aureus* bacteria (ATCC 25923 strain) treated with temporin-SHe. (**A**) Control: untreated *S. aureus* bacteria. (**B**) Effect of temporin-SHe (6.25 µM). (**C**) Effect of temporin-SHd (25 µM). The scale bar indicated on the right bottom represents 2 µm.

In agreement with the high activity (MIC = 3.12 µM) and potent bactericidal effect of temporin-SHe on *S. aureus*, we used scanning electron microscopy to visualize the Gram-positive bacterial architecture during peptide killing. The results showed disruption of the bacterial envelope structure after 30 min incubation with temporin-SHe (Figure 9B), suggesting damage caused by the peptide. In contrast, the control bacterial cell envelope architecture was unaffected (Figure 9A). Treatment with temporin-SHd resulted in the same bacterial damage, with protuberances and also cracks in the cell envelope (Figure 9C). On the basis of the cell envelope architecture, the estimated percentage of damaged bacteria was 78% and 82% for temporin-SHe 6.25 µM and temporin-SHd 25 µM, respectively.

## 3. Discussion

Temporin-SHe and temporin-SHd are two temporin-SH paralogs belonging to the long temporin subfamily that were identified in the skin of the Sahara Frog (*Pelophylax saharicus*) [8]. While, in general, the mean size of temporins is 13–14 amino acid residues, the long temporin subfamily contains very few members. Indeed, out of the 119 temporins currently listed in APD (http://aps.unmc.edu/AP/main.php) only 8 and 13 are 16-and 17-residue-long peptides, respectively. Since the identification of temporins SHe and SHd in 2010, only temporin-SHd was further characterized. The latter was demonstrated to be a broad-spectrum antibacterial and antiparasitic peptide [15].

We reported here, for the first time, the structural and functional characterization of temporin-SHe, the most closely related paralog of temporin-SHd. The analysis of the physicochemical properties revealed that, in addition to their similar length (16–17 amino acid residues), both peptides have an identical net charge (+2), with, however, a slightly more pronounced hydrophobicity for temporin-SHe (Table 1). They exhibited 76.5% identity and differ from each other by only a glycine residue in terms of amino acid composition. Circular dichroism spectroscopy studies demonstrated that temporin-SHe adopted a well-defined α-helical structure when bound to model membrane vesicles (Figure 1). When plotted on a Schiffer–Edmundson helical wheel (Figure 2), temporin-SHe presented a not well-defined amphipathic helical structure, with a highly hydrophobic face composed by bulky apolar residues (Ile, Leu, Phe) and a slightly more polar face. The latter is constituted of a lysine residue with dual polar/apolar properties (ε-amino group and hydrocarbon chain, respectively), and neutral/small apolar residues (Gly/Ala, Pro). Temporin-SHd and temporin-SHe thus share high hydrophobicity (<H> = 0.86−0.91) and low amphipathicity (<µH> = 0.52−0.56). Amphipathic helical conformation of AMPs is known to play an important role in the mechanism of action, where the charged polar face drives electrostatic attraction to the negatively charged membrane and then the apolar face interacts with the hydrophobic core, leading to membrane permeabilization and/or disruption [35,36].

Even with a low amphipathic character, the data obtained by differential scanning calorimetry indicated that temporin-SHe was able to strongly perturb the membrane of bacterial model multilamellar vesicles (MLVs). Firstly, the disappearance of the pretransition peak occurred, which is a consequence of the temporin-SHe interaction with phospholipid headgroups. Secondly, the main transition peak shifted to higher temperatures, indicating a deep penetration of the hydrophobic face of the temporin-SHe α-helix into the fatty acyl chains of the lipid bilayer. An increase in the peptide amount (peptide–lipid ratio 1:50) even led to a two-component main phase transition, resulting from two coexisting phases. This two-component main phase transition was also observed for temporin-SHd, previously demonstrated to strongly and selectively perturb the membrane of anionic DMPG MLVs [15], and was assigned to peptide-poor (high-temperature component) and peptide-rich (low-temperature component) phospholipid domains [37]. Previous studies of Epand and coworkers have shown that the clustering of positive charges around the axis of the helix was important to promote phase segregation and thus activity [38]. Temporin-SHe and temporin-SHd have a net charge of +2 but the positive charges (i.e., α-NH_3_^+^ terminal group and ε-NH_3_^+^ group of lysine) are distributed differently, being located much closer in temporin-SHd than in temporin-SHe (Figure 2). This may explain the better activity of temporin-SHd toward *E. coli* strains (MIC = 5–50 µM) compared to temporin-SHe (MIC = 25–50 µM). Indeed, when temporin-SHd is bound to membranes, its cationic groups can adapt to the distance between negative charges of membrane lipids and may therefore promote a more easily segregation of anionic and zwitterionic lipids, leading to membrane collapse. Even though calorimetric data shed light on this mechanism, fluorescence resonance energy transfer studies would probably provide additional evidence.

Temporin-SHe showed potent activity against various Gram-positive bacteria with MIC ranging from 1.56 to 12.5 µM, and also moderate activity against the Gram-negative *P. aeruginosa* and the *Candida* species *C. parapsilosis* that were not sensitive to temporin-SHd (Table 2). In order to investigate the bacterial mode of action of temporin-SHe, we analyzed the effect of this peptide on the bacterial cytoplasmic membrane (permeabilization/depolarization assay) and its ability to kill bacteria. Temporin-SHe has bactericidal effects against Gram-positive as well as Gram-negative bacteria, but it was revealed highly effective against *S. aureus* bacteria (rapid and complete killing within 5 min) compared to *E. coli* bacteria (90 min for complete killing). In addition, temporin-SHe was able to permeabilize/depolarize the bacterial membrane in a time- and concentration-dependent manner. Thus, bacterial killing is correlated to permeabilization of the cytoplasmic membrane. Interestingly, temporin-SHd at 12.5 µM (two-fold MIC concentration) was able to depolarize the cytoplasmic membrane of *S. aureus* but did not induce complete death of bacteria within 120 min, although it was previously demonstrated to permeabilize the membrane of *S. aureus* and to induce rapid (15 min) and complete killing of these bacteria at 20 μM (3 × MIC concentration) [15]. These results could be explained by the two-state model, where peptide has two physical states of binding to the lipid bilayer, one at low peptide/lipid ratio (P/L) and another at a high P/L. When a threshold ratio is reached, the peptide tends to form a stable multi-pore state, whereas the few pores formed below the threshold concentration are usually unstable [39]. Thereby, temporin-SHd could translocate by these transient pores and interact with intracellular components, delaying the killing effect. The membranolytic mechanism suggested by peptide-induced membrane permeabilization/depolarization was confirmed by scanning electron microscopy, revealing that temporin-SHe and temporin-SHd were able to cause disruption of the *S. aureus* membrane architecture (Figure 9). Interestingly, temporin-SHe, like temporin-SHd, was as active against clinical isolates of antibiotic-resistant and -multiresistant *S. aureus* (ATCC 43300 and ATCC BAA-44) as against sensitive *S. aureus* strains (ATCC 25923 and ST1065) (Table 2). The original and effective mode of action of these AMPs compared to conventional antibiotics indicate that temporins are promising candidates in the fight against antibiotic-resistant pathogens. Nonetheless, our results indicated that temporin-SHe was highly cytotoxic compared to temporin-SHd toward the human mammalian cells tested (erythrocytes and THP-1 monocytes). This can be explained by the slightly higher intrinsic hydrophobicity of temporin-SHe since it is known that increasing the hydrophobic character leads to more cytotoxic AMPs [40]. In this case, it will be interesting to modulate the hydrophobicity and amphipathicity properties of temporin-SHe in order to increase its therapeutic effectiveness [41,42].

Temporin-SHe represents an additional temporin with antiparasitic properties. This peptide was able to kill promastigote forms of the human parasite *Leishmania*, responsible for visceral (*L. infantum*), cutaneous (*L. major*) and muco-cutaneous (*L. braziliensis*) leishmaniasis. Compared to temporin-SHd, temporin-SHe exhibited higher antileishmanial activity (Table 3), particularly against *L. infantum*. Until today, only six temporins (Ta, Tb, SHa, SHd, Tl and Tf) have been described as antiparasitic peptides and very few AMPs of other families are active against parasites (The Antimicrobial Peptide Database, http://aps.unmc.edu/AP/main.php). Temporins Ta and Tb were shown to be active against *Leishmania donovani* promastigotes and *L. pifanoi* axenic amastigotes [18]. Temporins SHa and SHd were described as broad-spectrum antileishmanial peptides, being active against several species including *L. infantum* (also antimony resistant *L. infantum*, for temporin-SHa), *L. major*, *L. braziliensis* and *L amazonensis* promastigotes (also *L. tropica*, for temporin-SHd), and *L. infantum* axenic and intramacrophagic amastigotes [13,14,15]. Both peptides were active against other trypanosomatids, such as *T. brucei* and *T. cruzi* epimastigotes. In addition to temporins Ta, Tb and SHa, Eggimann and collaborators demonstrated activity against *Leishmania mexicana* promastigotes for temporins Tl and Tf, but only temporin-SHa and temporin-Tl were active against *L. mexicana* amastigotes with however a less efficiency (approximately 10-fold and 17-fold, respectively) [19]. The mechanism by which temporins exert their antiparasitic activity is not fully understood. Both temporin-SHa and temporin-SHd exhibited intracellular leishmanicidal activity and were able to kill *L. infantum* amastigotes into macrophage cells, with higher activity compared to extracellular forms (promastigotes and axenic amastigotes) [14,15]. The leishmanicidal activity of temporin-SHa was demonstrated to occur via a primary membranolytic mechanism but also via other cell death mechanisms (apoptotic-like death). [14]. This intracellular killing ability of temporins was also observed on bacteria. Di Grazia and collaborators showed that temporin-Tb, and also temporin-Ta to a lesser extent, were able to kill *S. aureus* bacteria (ATCC 25923 and MRSA strains) within infected HaCaT keratinocytes [43]. The similar killing of MRSA cells inside HaCaT keratinocytes was also observed for an analog of temporin-CEb (formerly temporin-1CEb) conjugated with dalargin, a Leu-enkephalin analog [44]. We have recently confirmed the intracellular activity of temporin-SHa, but this time against a bacterial pathogen, the Gram-negative *Legionella pneumophila* responsible for Legionnaire’s disease [31]. Temporin-SHa killed *L. pneumophila* bacteria within both amoebae and macrophages.

A search in the database Antimicrobial Peptide Database (APD) allowed us to find the five AMPs most similar to temporin-SHe. Not surprisingly, these AMPs belong to the temporin family. In addition to temporin-SHd that shares 76.5% identity, the four other AMPs displayed 61–68.7% identity with temporin-SHe. These peptides correspond to peptide B9 (75% identity), temporin-HB2 (68.7%), temporin-1Ec (62.5%), and temporin-TP3 (61.1%). Peptide B9 (FLPLIAGLLGKLF_amide_) is a potent hemolytic peptide (100% hemolysis of human erythrocytes at 4.5 µM) isolated by Simmaco and collaborators from skin extracts of *Rana esculenta* (reclassified now as *Pelophylax lessonae*/*ridibundus*), and predicted to be structured as an amphipathic α-helix [45]. This peptide was further considered as a member of the temporin family after the identification of temporin-1Ec sharing 84.6% identity with B9 [46], and reflections on the nomenclature of AMPs from the frogs of the family Ranidae [47]. Temporin-1Ec (FLPVIAGLLSKLF_amide_), which was also isolated from *Pelophylax lessonae*/*ridibundus* (formerly *R. esculenta*), was shown to be active against *S. aureus* (MIC = 8 µM) but not against *E. coli* (MIC > 100 µM) [46]. Temporin-HB2 (FLPFLAGLFGKIF_amide_) was isolated from the Hubei Frog *Pelophylax hubeiensis* [48]. This hemolytic peptide (65% hemolysis of human erythrocytes at 25 µM) demonstrated activity against the Gram-positive bacteria *E. faecalis* (MIC = 19 µM) and low activity against the Gram-positive *Nocardia asteroides* (MIC = 75 µM), but was not active against *S. aureus* (MIC = 150 µM). No activity was found against Gram-negative bacteria (*E. coli*, *P. aeruginosa*, and *K. pneumoniae*) and *Candida* species (*C. albicans* and *C. glabrata*) [48]. Like temporin-HB2, temporin-TP3 (FLPLLFGALSTLLPKIF_amide_), isolated from skin secretions of *Hylarana taipehensis*, is hemolytic (51% hemolysis at 6.3 µM) and has an antimicrobial spectrum restricted to Gram-positive bacteria, with MICs determined as follows: 25 µM for *S. aureus*, 50 µM for *E. faecalis*, and 12.5 µM for *N. asteroides* [49]. Therefore, on the basis of the activity of the five AMPs most similar to temporin-SHe, only temporin-SHd shares with temporin-SHe a broad-spectrum antimicrobial activity.

In conclusion, temporin-SHe represents a new potent broad-spectrum antibacterial and antiparasitic temporin-SH paralog, in addition to the temporins SHa and SHd that were previously characterized. The effectiveness of temporin-SHe against resistant pathogens makes this peptide an attractive candidate, which however needs modifications due to its cytotoxicity to design optimized analogs with therapeutic potential. Nevertheless, as an additional useful tool, temporin-SHe should help to decipher the antiparasitic mechanism of temporins, and more generally, of AMPs.

## 4. Materials and Methods

### 4.1. Peptide Synthesis

Synthesis of carboxyamidated temporin-SHe was performed using a solid-phase FastMoc chemistry procedure on a 433A automated peptide synthesizer from Applied Biosystems, as previously described [50]. Briefly, Fmoc-Rink-Amide PEG MBHA resin and Fmoc-protected amino acids were purchased from Iris Biotech GMBH (Marktredwitz, Germany). Purification was performed by reversed-phase high-performance liquid chromatography (RP-HPLC) on a semi-preparative column (Luna C18, 10 µm, 250 × 10 mm, Phenomenex, Torrance, CA, USA) with a 40–80% linear gradient of acetonitrile (1%/min) at a flow rate of 5 mL/min. Peptide purity was assessed by analytical RP-HPLC on an Uptisphere C18 column (modulo-cart QS, 5 μm, ODS2, 250 × 4.6 mm, Interchim, Los Angeles, CA, USA) using the conditions above with a flow rate of 0.75 mL/min. The peptide mass was confirmed by MALDI-TOF-MS (Voyager DE-Pro and 4700 Proteomic analyzer, Applied Biosystems, Mass Spectrometry platform, IBPS, Sorbonne Université, France). Carboxamidated temporin-SHd and [K^3^]temporin-SHa, also used in the study, were synthesized using the same procedure. The figures of HPLC chromatograms (Figures S1–S3) and MS spectra (Figures S4–S6) of the synthesized peptides were provided as Supplementary Data. 

### 4.2. Conformational Study

The secondary structure of temporin-SHe was determined by circular dichroism (CD) in membrane-mimicking environments. CD measurements were performed as described [15] in phosphate buffer (10 mM Na_2_HPO_4_, pH 7.3) containing negatively charged DMPC/DMPG (3:1) LUVs (bacterial membrane model), at a peptide/lipid molar ratio of 1:100. CD spectra of temporin-SHe (30 µM) were also obtained in PBS alone and in PBS containing 80 mM sodium dodecyl sulfate (SDS micelles). DMPC, dimyristoyl phosphatidylcholine; DMPG, dimyristoyl phosphatidylglycerol. Lipids were purchased from Avanti Polar Lipids, Inc. (Alabaster, AL, USA).

### 4.3. Differential Scanning Calorimetry

Differential scanning calorimetry experiments were performed using DMPC/DMPG 3:1 multilamellar vesicles (MLVs) and different peptide/lipid molar ratios (1:200, 1:100 and 1:50), according to the procedure described in [8]. Several scans (>20) were run for each sample with a 10 min equilibration time between each scan. The raw data were analyzed with the CpCalc software and thermodynamic values (T_m_ and ΔH) were estimated by a peak-fitting procedure.

### 4.4. Microorganisms and Cells

Antibacterial activity of temporin-SHe was evaluated against Gram-positive bacteria including *S. aureus* strains (ATCC 25923, ST1065), antibiotic-resistant *S. aureus* strains (ATCC 43300, ATCC BAA-44), *E. faecalis* (ATCC 29212), *B. megaterium*, and *L. ivanovii* (Li4pVS2), and Gram-negative bacteria including *E. coli* strains (ATCC 25922, ATCC 35218, ML-35p), *S. enterica* (serotype Enteritidis), *P. aeruginosa* (ATCC 27853), *A. baumannii* (ATCC 19606) and *K. pneumoniae* (ATCC 13883). Antifungal activity was evaluated against *C. albicans* (ATCC 90028), *C. parapsilosis* (ATCC 22019) and *S. cerevisiae*. ATCC strains were purchased from American-Type Culture Collection (ATCC, Virginia, VA, USA). Antiparasitic activity was determined against promastigotes of several species of *Leishmania* responsible for visceral, cutaneous and mucocutaneous leishmaniases: *L. infantum* (strain MHOM/MA/67/ITMAP-263), *L. major* (strain MHOM/SU/73/5-ASKH), and *L. braziliensis* (strain MHOM/BR/75/M2904), respectively. Cytotoxicity was assessed against human red blood cells and THP-1 monocytes. Peripheral blood was obtained from healthy adult donors (Établissement Français du Sang, Paris, France). All donors signed informed consent allowing the use of their blood for research purposes.

### 4.5. Antibacterial and Antifungal Activities

Antimicrobial activity was assessed with a liquid growth inhibition assay performed according to a previously described protocol [14] using the Clinical and Laboratory Standards Institute (CLSI) guidelines. Briefly, in 96-well microtitration plates, mid-log-phase bacteria diluted to 10^6^ CFU/mL in Mueller–Hinton (MH) broth were added to different concentrations of synthetic temporin-SHe (1–200 µM, final concentrations) and incubated at 37 °C for 18–20 h, under shaking (150 rpm). For *E. faecalis*, *L. ivanovii* and yeasts/fungi, 10^6^ CFU/mL suspensions were prepared in LB broth, brain heart infusion (BHI) medium and Yeast Peptone Dextrose (YPD) medium, respectively, and incubation was performed at 30 °C for yeasts/fungi. After incubation, the optical density was evaluated at 630 nm and the minimal inhibitory concentration (MIC), corresponding to the lowest concentration of peptide that totally inhibited bacterial growth, was determined. MIC values represent the average of three independent experiments, each performed in triplicate with negative (no peptide) and positive (formaldehyde 0.7%) controls.

### 4.6. Antiparasitic Activity

Antileishmanial activity of temporin-SHe was analyzed by a luminescence-based growth inhibition method using *Leishmania* promastigotes transfected with the vector pGMαNEOαLUC containing the gene LUC that codes for the firefly luciferase, cultured at 26 °C in SDM-79 medium supplemented with 10% fetal calf serum (Gibco), as previously described [14]. Mid-log-phase promastigotes (1.25 × 10^6^ cells/mL) were added in 96-well plates containing different concentrations of synthetic temporin-SHe (3.125–50 µM, final concentrations), and incubated 72 h at 26 °C. After incubation, the luciferase activity was revealed by using Steady-Glo^®^ Assay System (Promega, Madison, WI, USA) and the 50% inhibitory concentration (IC_50_) was determined. IC_50_ values represent the average of three independent experiments, each performed in triplicate. IC_50_ was determined with GraphPad Prism^®^ 6.0 software (GraphPad Software, La Jolla, CA, USA) using a sigmoidal dose–response curve fitting equation.

### 4.7. Cytotoxic Activity

Hemolytic activity of temporin-SHe was determined against human red blood cells (RBCs), as described in [13]. After incubating the peptide (1–200 µM, final concentrations) with RBCs (2 × 10^7^ cells) in 100 µL PBS for 1 h at 37 °C, centrifugation was completed (12,000× *g*, 15 s) and the absorbance of the supernatant was measured at 450 nm. RBCs suspended in PBS or 0.1% (*v*/*v*) Triton X-100 yielded an absorbance associated with 0% and 100% hemolysis, respectively. The percentage of hemolysis was calculated as follow: Hemolysis (%) = [A_450_ (RBCs with peptide)–A_450_ (RBCs in PBS)]/[A_450_ (RBCs with Triton X-100)–A_450_ (RBCs in PBS)]. Cytotoxicity of temporin-SHe was also assessed on human leukemia monocyte cell line THP-1. Briefly, monocytes (6.25 × 10^5^ cells/mL) were incubated with different peptide concentrations (3–50 µM, final concentrations) for 72 h at 37 °C, then cell viability was determined by the MTT assay according to a previously described protocol [50]. Results were expressed as the mean of two independent experiments performed in triplicate.

### 4.8. Membrane Permeabilization Assay

The ability of temporin-SHe to permeabilize bacterial cytoplasmic membrane was determined using the Gram-negative strain *E. coli* ML-35p and the Gram-positive strain *S. aureus* ST1065, as described [8,15]. Briefly, after incubation of bacteria (37 °C) in 96-well plates with PBS containing 2.5 mM o-nitrophenyl-β-d-galactopyranoside (ONPG) and different concentrations of temporin-SHe, the production of o-nitrophenol (ONP) resulting from hydrolysis of ONPG by intracytoplasmic β-galactosidase was monitored at 405 nm according to the time (Fluostar Galaxy plate reader, BMG Labtech, Champigny-sur-Marne, France). Wells containing bacteria and ONPG, with no peptide, were used as negative control. Two independent experiments were performed in triplicate. Results are from a representative experiment and were expressed as the mean ± S.D.

### 4.9. Membrane Depolarization Assay

The depolarization of *S. aureus* ATCC 25923 cytoplasmic membrane induced by temporin-SHe and temporin-SHd was investigated using the membrane potential-sensitive cyanine dye DiSC_3_(5) (3,3′-dipropylthiadicarbocyanine iodide), as detailed in [29]. An amount of 700 µL of bacteria resuspended in PBS (10^7^ cfu/mL) containing 1 μM DiSC_3_(5) were preincubated in the dark during 10 min at 37 °C, and then 100 µL of 1 mM KCl were added to the mixture. Fluorescence (λex = 622 nm; λem = 670 nm) was recorded during 20 min at 37 °C (Varian Cary Eclipse fluorescence spectrophotometer) after addition of the peptide (200 µL, final concentration: 2-fold above the MIC). Three independent experiments were performed and the results correspond to a representative experiment with negative (PBS) and positive (melittin) controls.

### 4.10. Time-Killing Assay

Time-killing kinetics of temporin-SHe and temporin-SHd against *S. aureus* ST1065 and *E. coli* ATCC 25922 were evaluated as previously described [15]. The peptides were incubated at 2-fold MIC with the bacterial cells (10^6^ cfu/mL) resuspended in PBS buffer. Aliquots were withdrawn at different times and spread onto LB agar plates for cell counting after overnight incubation at 37 °C. Controls corresponding to the bacterial suspensions without peptide were also run. Two independent experiments were performed in triplicate. Results were expressed as the mean ± S.D. of a representative experiment.

### 4.11. Scanning Electron Microscopy (SEM) Imaging

We used the Gram-positive species *S. aureus* ATCC 25923 to visualize the effects of temporin-SHe and temporin-SHd on the bacterial membrane. Bacteria were cultivated overnight in MH broth at 37 °C under agitation (250 rpm). After centrifugation (10,000× *g*, 5 min), bacterial cells were harvested and dispersed in an isotonic sterile solution (NaCl 0.9%) to obtain a density of 2 × 10^7^ cfu/mL. The bacterial suspension was incubated with the peptide during 30 min onto sterile stainless steel 1 cm × 1 cm surfaces. After this time, the surface was washed 3 times with PBS to remove all non-adhering bacteria and then fixed with 2.5% glutaraldehyde to avoid collapsing of cells upon drying. SEM images were recorded with a ThermoFisher low vacuum Apreo S field emission gun scanning electron microscope. The samples were fixed on an alumina SEM support with a carbon adhesive tape and were observed without metallization. In-lens secondary electron detector was used in standard mode (Everhart Thornley Detector, ETD) to detect only secondary electrons. The accelerating voltages were comprised between 3 and 5 kV, and the working distance was around 10 mm. At least ten different locations were analyzed on each surface, arising to the observation of a minimum of 100 single bacteria observed.

### 4.12. Statistical Analyses

Statistics were determined with GraphPad Prism^®^ 6.0 software (GraphPad Software, La Jolla, CA, USA). Data are represented as mean ± S.D. A one-way ANOVA followed by a Dunnett test or by a Tukey multiple comparison test was performed for comparison between the control and treated cells or for comparison between each condition (* *p* < 0.05; ** *p* < 0.01; *** *p* < 0.001).

## Figures and Tables

**Table 1:** Sequence alignment and physicochemical properties of temporins-SH.

Temporin	Sequence Alignment ^1^	Reference	Residue	Net Charge ^2^	Mw ^3^	GRAVY ^4^
SHa	FLSGIVGMLGKLF_amide_	[13,14,23]	13	+2	1381.74	1.67
SHb	FLPIVTNLLSGLL_amide_	[13,23]	13	+1	1399.74	1.81
SHc	FLSHIAGFLSNLF_amide_	[13,23,24]	13	+1	1465.71	1.34
SHd	FLPAALAGIGGILGKLF_amide_	[15]	17	+2	1658.06	1.65
SHe	FLP-ALAGIAGLLGKIF_amide_	[8]	16	+2	1601.01	1.78
SHf	FFFLSRIF_amide_	[8]	8	+2	1076.31	1.77

^1^ ClustalW alignment (https://npsa.lyon.inserm.fr/). Identical amino acids are highlighted in grey.^2^ The peptide net charge is at neutral pH. ^3^ Molecular weight and ^4^ Grand average of hydropathicity were calculated using ProtParam (https://web.expasy.org/protparam/).

**Table 2:** Antimicrobial activity of temporin-SHe compared to temporin-SHd.

	MIC (µM) ^1^	
	Temporin-SHe	Temporin-SHd
**Gram-negative bacteria**		
*E. coli* ATCC 25922	25	5 *
*E. coli* ATCC 35218	50	50 *
*E. coli* ML-35p	50	25 *
*P. aeruginosa* ATCC 27853	60	>200 *
*S. enterica* ^2^	100	>200 *
*A. baumannii* ATCC 19606	25	25 *
*K. pneumoniae* ATCC 13883	100	100 *
**Gram-positive bacteria**		
*S. aureus* ATCC 25923	3.12	6.25 *
*S. aureus* ATCC 43300 ^3^	3.12	6.25 *
*S. aureus* ATCC BAA-44 ^4^	3.12	6.25 *
*S. aureus* ST1065	3.12	6.25 *
*L. ivanovii*	5	10
*E. faecalis* ATCC 29212	12.5	25 *
*B. megaterium*	1.56	1.56 *
**Yeasts/fungi**		
*C. albicans* ATCC 90028	>100	100 *
*C. parapsilosis* ATCC 22019	50	>200 *
*S. cerevisiae*	12.5	25 *

^1^ Minimal inhibitory concentration. ^2^ *Salmonella enterica* serotype Enteritidis. ^3^ Resistant to methicillin and oxacillin. ^4^ Resistant to amoxicillin/clavulanic acid, cephalothin, ciprofloxacin, erythromycin, gentamicin, imipenem, oxacillin, penicillin, tetracycline, ampicillin, doxycycline, methicillin, azithromycin, ceftriaxone, clindamycin, lincomycin, perfloxacin, rifampin and tobramycin. * Values taken from [15].

**Table 3:** Activity of temporin-SHe on different *Leishmania* species.

	IC_50_ (µM) ^1^	
	Temporin-SHe	Temporin-SHd
***L. infantum***	4.6	16.5 *
***L. braziliensis***	10.5	17.9 *
***L. major***	11.6	14.6 *

^1^ IC_50_ values (half-maximal inhibitory concentrations) represent the mean of three independent experiments performed in triplicate. * Values were taken from reference [15].

## Supplementary Materials

The following are available online at https://www.mdpi.com/1422-0067/21/18/6713/s1, Figure S1: Analytical RP-HPLC of the synthesized temporin-SHe. Figure S2: Analytical RP-HPLC of the synthesized temporin-SHd. Figure S3: Analytical RP-HPLC of the synthesized [K^3^]temporin-SHa. Figure S4: MALDI-TOF MS spectrum of the synthetic temporin-SHe. Figure S5: MALDI-TOF MS spectrum of the synthetic [K^3^]temporin-SHa. Figure S6: MALDI-TOF MS spectrum of the synthetic temporin-SHd.
