# Supplementary material for: Functional Characterization of Temporin-SHe, a New Broad-Spectrum Antibacterial and Leishmanicidal Temporin-SH Paralog from the Sahara Frog (Pelophylax saharicus)
Source: Int J Mol Sci. 2020 Sep 13;21(18):6713. doi: 10.3390/ijms21186713 (PMC7555312; doi:10.3390/ijms21186713)
Supplement: Supplementary file 1 [file ijms-21-06713-s001.pdf]

Manuscript ID: ijms-910693

Supplementary Figures of the revised version of manuscript ijms-910693

Functional Characterization of Temporin-SHe, a New Broad-Spectrum Antibacterial and Leishmanicidal Temporin-SH Paralog from the Sahara Frog (Pelophylax saharicus)

By André et al.

A

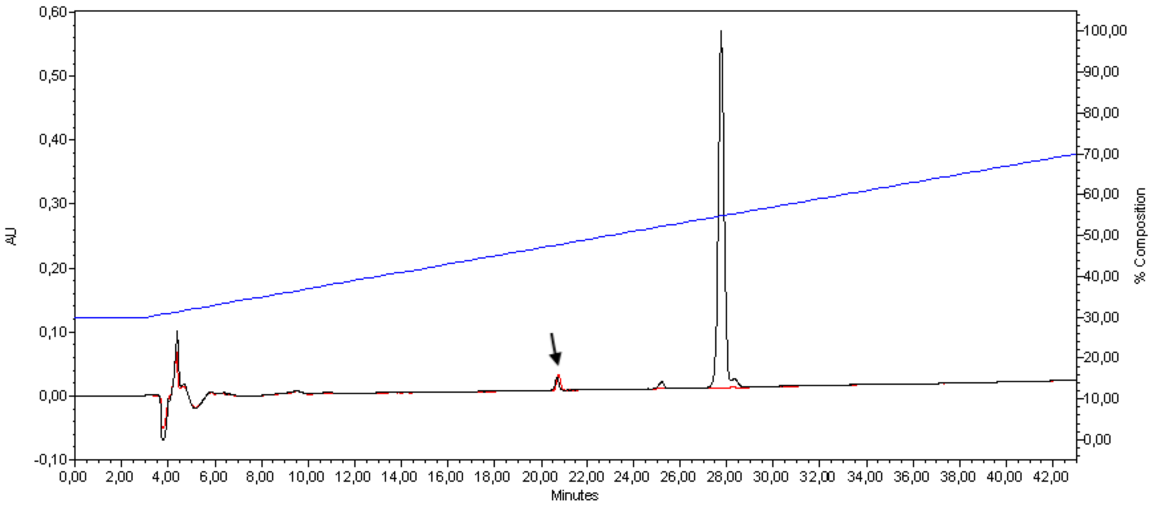

B

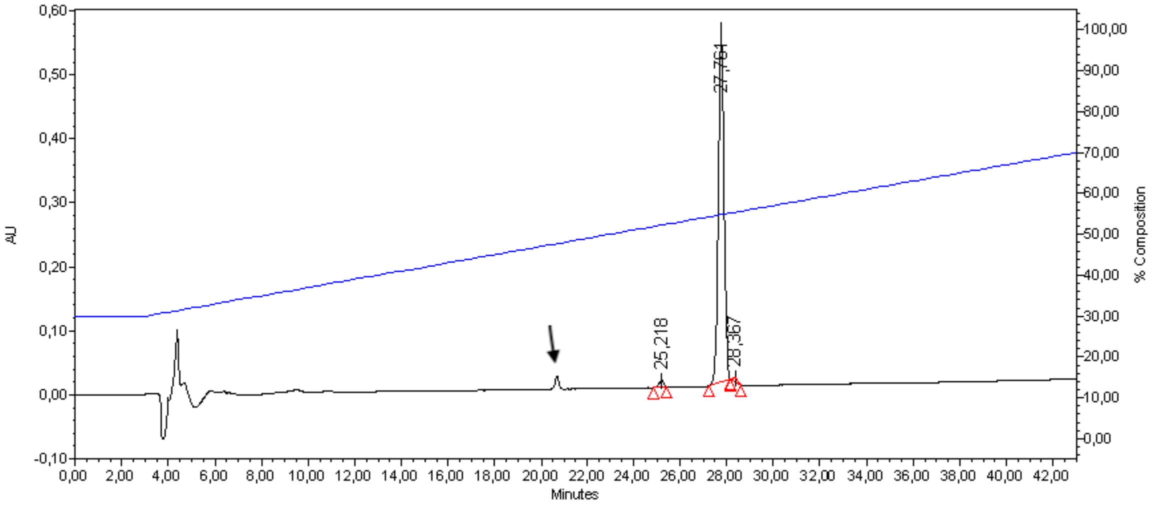

Retention Time	Area	% Area	Height	% Height
25.218	162082	1.72	10479	1.85
27.761	9192709	97.37	551041	97.06
28.367	86061	0.91	6197	1.09

Figure S1. Analytical RP-HPLC of the synthesized temporin-SHe performed on a C18 column with a 30-70% linear gradient of acetonitrile (1%/min, see % composition axis) (blue line). Absorbance was monitored at 220 nm (AU: absorbance unit). (**A**) HPLC chromatogram showing elution of temporin-SHe at a retention time corresponding to 27.76 min (black line). Blank chromatogram (run without peptide) is also shown ('red line) and revealed a small peak eluting at t = 20.7 min (arrow). (**B**) Peak integration indicating 97.4% peptide purity (% area). The peak denoted by an arrow was not integrated as it was also present in the blank.

A

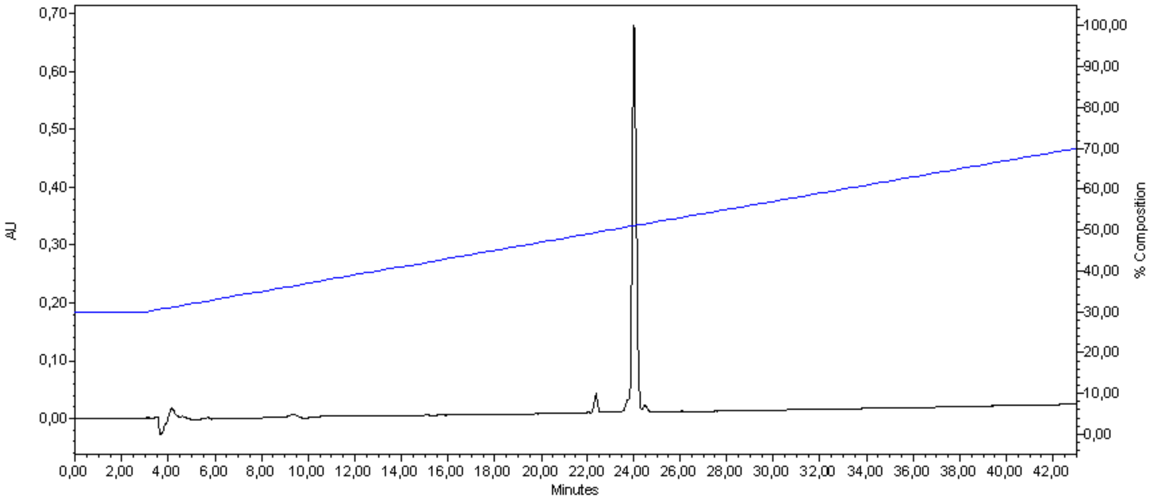

B

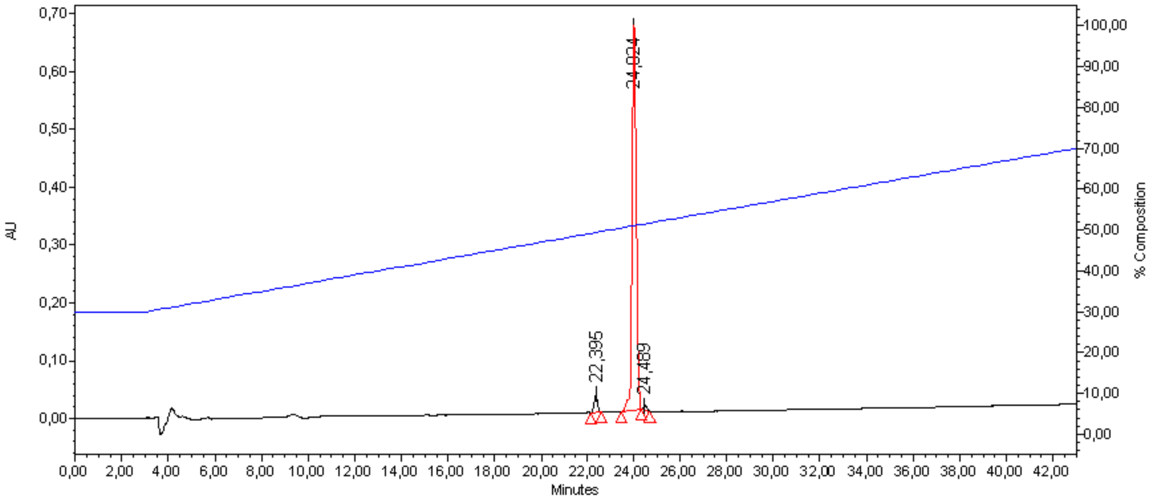

Retention Time	Area	% Area	Height	% Height
22.395	307062	3.59	32293	4.57
24.024	8164956	95.43	666509	94.37
24.489	83997	0.98	7468	1.06

Figure S2. Analytical RP-HPLC of the synthesized temporin-SHd performed on a C18 column with a 30-70% linear gradient of acetonitrile (1%/min, see % composition axis) (blue line). Absorbance was monitored at 220 nm (AU: absorbance unit). (**A**) HPLC chromatogram showing elution of temporin-SHd at 24.02 min. (**B**) Peak integration indicating 95.4% peptide purity (% area).

A

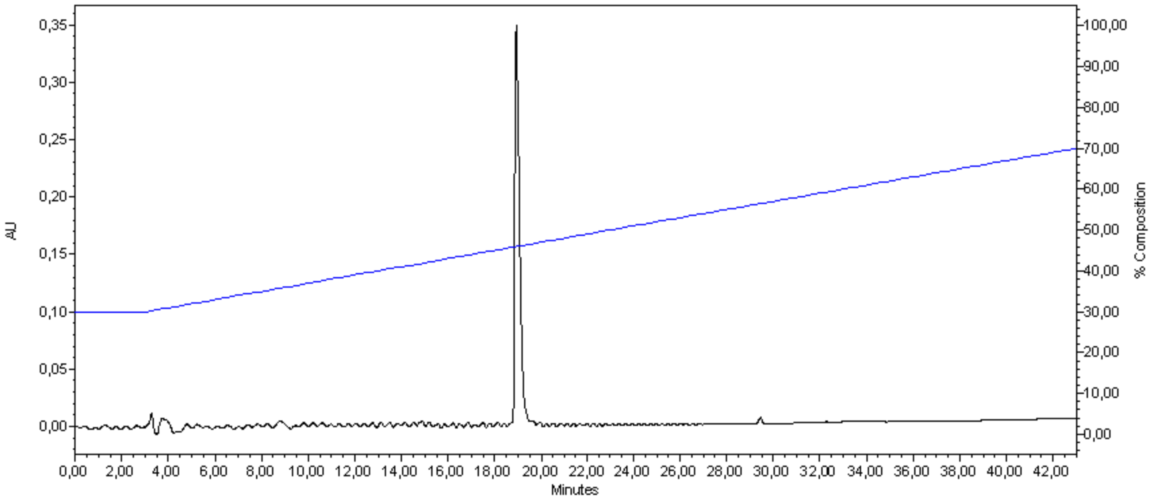

B

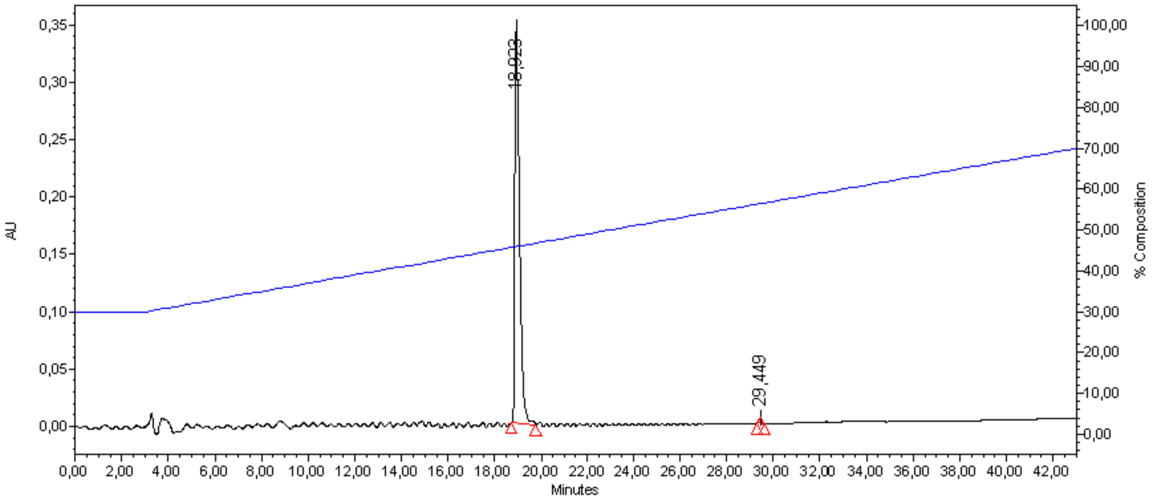

Retention Time	Area	% Area	Height	% Height
18.923	4980943	99.14	346831	98.57
29.449	43043	0.86	5040	1.43

Figure S3. Analytical RP-HPLC of the synthesized [K^3]temporin-SHa performed on a C18 column with a 30-70% linear gradient of acetonitrile (1%/min, see % composition axis) (blue line). Absorbance was monitored at 220 nm (AU: absorbance unit). (**A**) HPLC chromatogram showing elution of [K^3]temporin-SHa at a retention time corresponding to 18.92 min. (**B**) Peak integration indicating 99% peptide purity (% area).

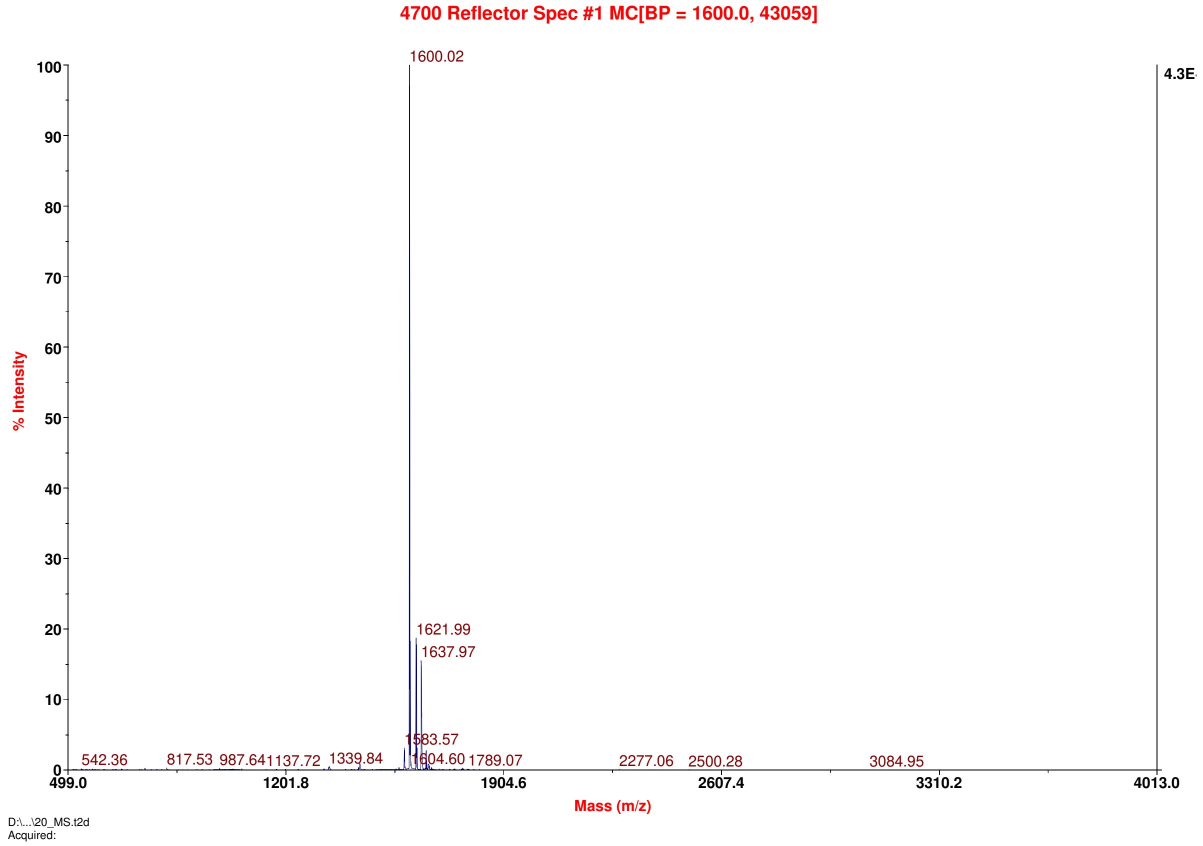

Figure S4. MALDI-TOF MS spectrum of the synthetic temporin-SHe. A mass product ($[M+H]^+$, 1600.02 *m*/*z*) corresponding to the theoretical monoisotopic mass of temporin-SHe is observed, with also the sodium and potassium adducts ($[M+Na]^+$, 1621.99 *m*/*z*; $[M+K]^+$, 1637.97 *m*/*z*).

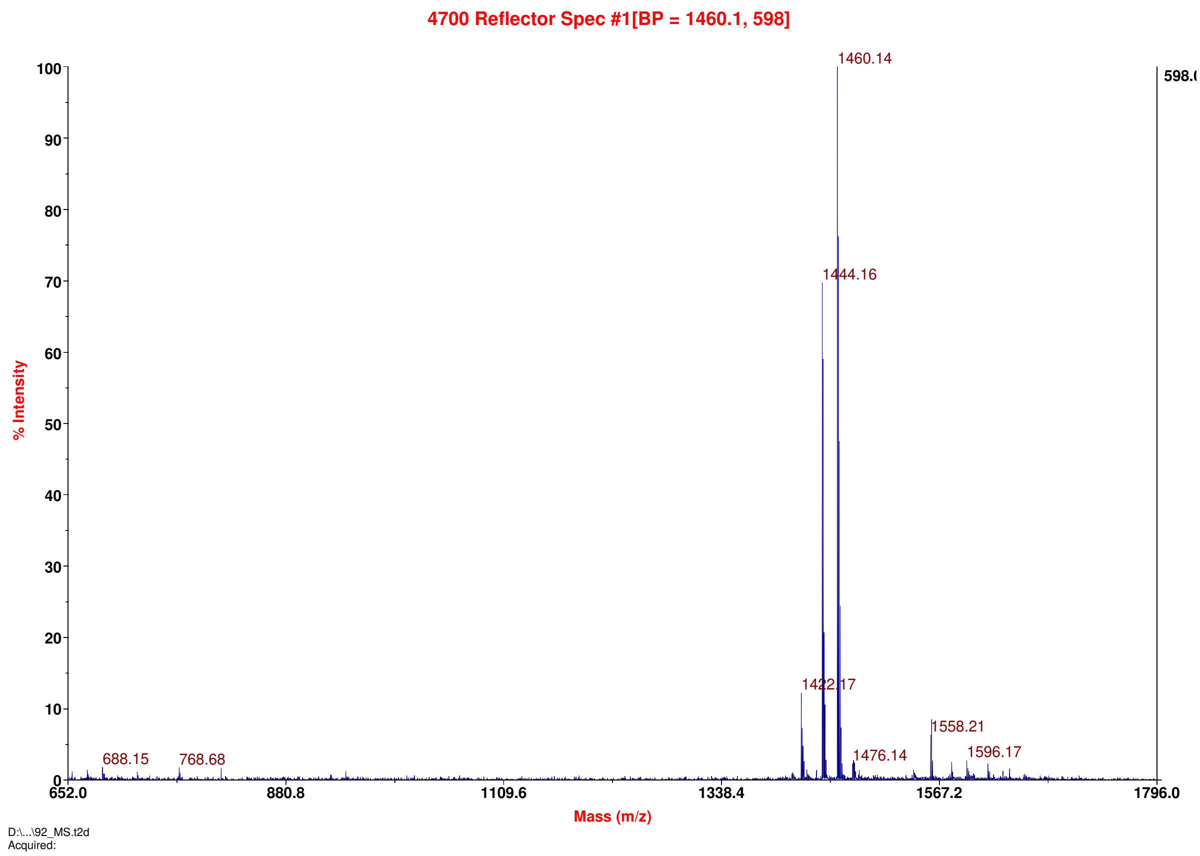

Figure S5. MALDI-TOF MS spectrum of the synthetic [K[3]]temporin-SHa. A mass product ([M+H]$^+$, 1422.17 *m*/*z*) corresponding to the theoretical monoisotopic mass of [K[3]]temporin-SHa is observed. Sodium and potassium adducts ([M+Na]$^+$, 1444.16 *m*/*z*; [M+K]$^+$, 1460.14 *m*/*z*) are also visible.

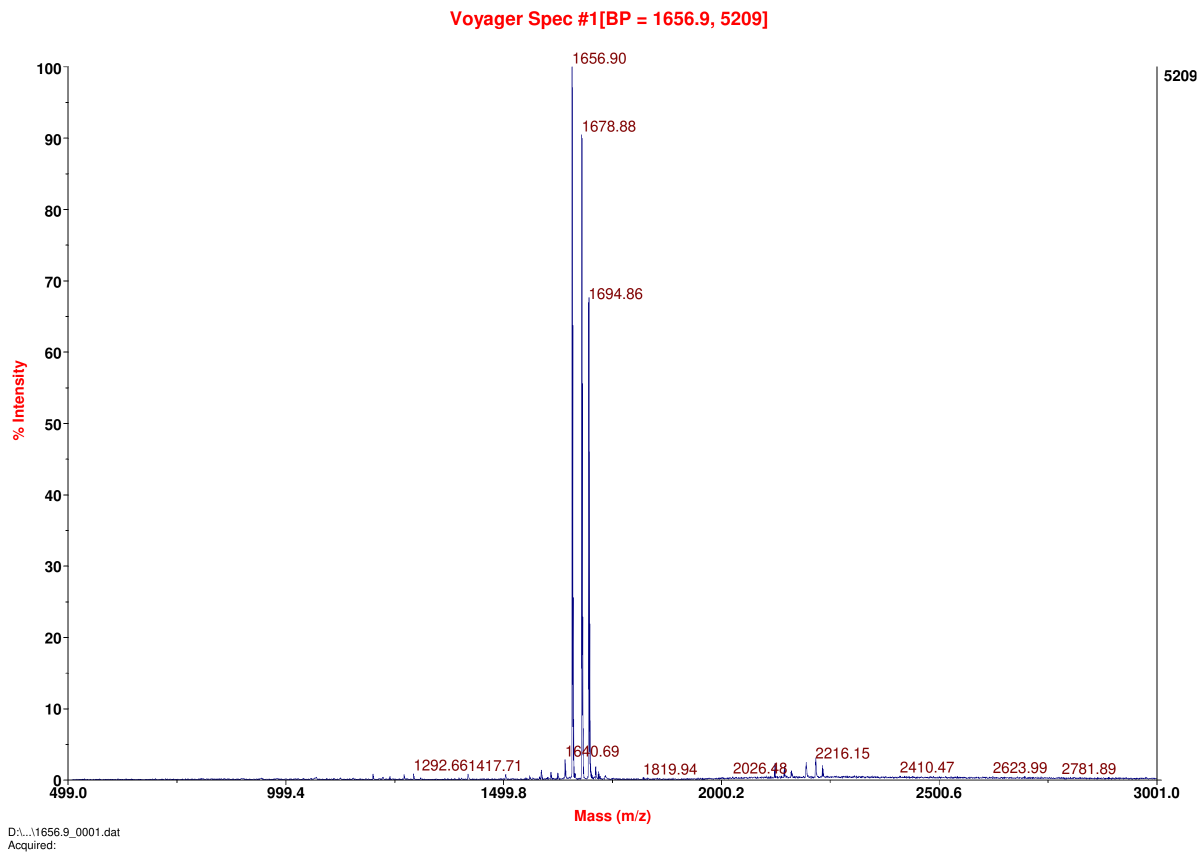

Figure S6. MALDI-TOF MS spectrum of the synthetic temporin-SHd. A mass product ($[M+H]^+$, 1656.90 *m/z*) corresponding to the theoretical monoisotopic mass of temporin-SHd is observed. Sodium and potassium adducts ($[M+Na]^+$, 1678.88 *m/z*; $[M+K]^+$, 1694.86 *m/z*) are also visible.
